# Single-trial extraction of event-related potentials (ERPs) and classification of visual stimuli by ensemble use of discrete wavelet transform with Huffman coding and machine learning techniques

**DOI:** 10.1186/s12984-023-01179-8

**Published:** 2023-06-02

**Authors:** Hafeez Ullah Amin, Rafi Ullah, Mohammed Faruque Reza, Aamir Saeed Malik

**Affiliations:** 1 School of Computer Science, Faculty of Science and Engineering, University of Nottingham, Jalan Broga, 43500 Semenyih, Malaysia; 2 Department of Computer and Information Sciences, Universiti Teknologi PETRONAS, 32610 Seri Iskandar, Malaysia; 3 Department of Neurosciences, School of Medical Sciences, Hospital Universiti Sains Malaysia, Kubang Kerian, 16150 Kota Bharu, Malaysia; 4 Faculty of Information Technology, Brno University of Technology, Brno, Czech Republic

**Keywords:** Single trials analysis (ERPs), Visual object detection, Discrete wavelet transform, Huffman coding, Machine learning classifiers

## Abstract

**Background:**

Presentation of visual stimuli can induce changes in EEG signals that are typically detectable by averaging together data from multiple trials for individual participant analysis as well as for groups or conditions analysis of multiple participants. This study proposes a new method based on the discrete wavelet transform with Huffman coding and machine learning for single-trial analysis of evenal (ERPs) and classification of different visual events in the visual object detection task.

**Methods:**

EEG single trials are decomposed with discrete wavelet transform (DWT) up to the $4{th}$ level of decomposition using a biorthogonal B-spline wavelet. The coefficients of DWT in each trial are thresholded to discard sparse wavelet coefficients, while the quality of the signal is well maintained. The remaining optimum coefficients in each trial are encoded into bitstreams using Huffman coding, and the codewords are represented as a feature of the ERP signal. The performance of this method is tested with real visual ERPs of sixty-eight subjects.

**Results:**

The proposed method significantly discards the spontaneous EEG activity, extracts the single-trial visual ERPs, represents the ERP waveform into a compact bitstream as a feature, and achieves promising results in classifying the visual objects with classification performance metrics: accuracies 93.60$\pm {6.5}$, sensitivities 93.55$\pm {4.5}$, specificities 94.85$\pm {4.2}$, precisions 92.50$\pm {5.5}$, and area under the curve (AUC) 0.93$\pm {0.3}$ using SVM and *k*-NN machine learning classifiers.

**Conclusion:**

The proposed method suggests that the joint use of discrete wavelet transform (DWT) with Huffman coding has the potential to efficiently extract ERPs from background EEG for studying evoked responses in single-trial ERPs and classifying visual stimuli. The proposed approach has *O(N)* time complexity and could be implemented in real-time systems, such as the brain-computer interface (BCI), where fast detection of mental events is desired to smoothly operate a machine with minds.

## Introduction

Investigation of the neural mechanism of the human brain through non-invasive imaging modalities is the primary goal of neuroscientists. Electroencephalography (EEG) is a neuroimaging technique that is most commonly used as a non-invasive and cost-effective imaging modality in the research community. The utmost prominent field of study in which the use of the EEG technique is applied and reported its usefulness is event-related potentials (ERPs). In an ERP study, the stimuli (events) presentation is synchronized with brain responses through an EEG acquisition device. When an event occurs, or a stimulus presents to the participant, it triggers responses to specific cognitive, sensory, or motor regions that can be collected through the EEG technique over the scalp. ERPs are useful in studying cognitive processes, clinical applications, and developing brain-computer interface (BCI) systems [1]. A standard method to extract an ERP signal is to take an average on the entire set of EEG segments, which have recorded the evoked potentials against the visual or auditory events (also known as trials). The trials are time-locked EEG recordings that are made synchronized with the neural activity occurring inside the brain during an experimental setup. In addition, averaging cancels out the noise as well as other ongoing neural processes [2]. The common assumption for this approach is that the human brain continuously behaves exactly in the same manner to specific stimulation. However, this may not be true as habituation and attention can influence brain responses [3]. The analysis of brain dynamics with the average ERPs method is well-known to researchers. Presently, the single trials of ERPs analysis have become a new interest with additional challenges [4]. The trial-to-trial variability is the limiting factor to getting high classification results between different types of events. Besides, single-trial responses are a mixture of task-related responses with tasks unrelated responses, which lowers the signal-to-noise ratio of the observed ERPs. The trial-to-trial variability within the subject or inter subjects variability is present in amplitudes and latencies of the ERP signals [5]. Thus, it is recommendable to enhance the feature extraction process of the ERP signals before applying any type of classification algorithm [6]. Different feature extraction methods have been developed to discriminate the ERP signals from noise, including spatial and temporal filters, e.g., bandpass filter, notch filter, principal component analysis (PCA), and more advanced de-noising techniques, such as wavelet de-noising and blind source separation techniques [7].

Previous studies on ERP extraction reported the use of features extracted from the temporal, frequency, and spatial domain to separate trials belonging to certain categories based on a single trial basis. The steps in signal processing include pre-processing, spatial filtering, feature extraction, and modeling with machine-learning classifiers [6]. The pre-processing of EEG for time-locked signals employs a band-pass filter with a lower cut-off frequency from 0.1 to 0.5Hz and an upper cut-off frequency of  30Hz [8]. The band-pass filtering removes unwanted signals, such as high-frequency artifacts and DC components, and retains the desired range of frequencies where the event-related potential signal can be extracted. The EEG signal can then be segmented using the time-stamped information of stimuli onset and offset. The segments include a portion of the signal before the stimulus onset, such as 100ms pre-stimulus, as a baseline line and the whole duration of the stimulus presentation until offset. Some individual segments may be contaminated by eye blinks and eye movements, i.e., if the amplitude exceeded $\pm 90\mu$V[9], which would either be discarded from further analysis or can be corrected by employing methods, such as ICA [10]. The segments (trials) can be visualized to detect those electrodes that may have lost contact in the event of widespread drift, as well as bad channels in the segments, spherical spline method is a good choice to correct bad channels [11]. Finally, the pre-processing step includes a data-independent spatial filtering technique called an averaged reference, which re-reference the data from the original single electrode used during data recording [12]. After the preprocessing, the next step in signal processing is feature extraction, which mines stimulus-related information from the pre-processed signals. The well-known methods of feature extraction include time, frequency, and time-frequency features.

In the literature on visual object classification based on single-trial ERP, many studies have focused on extracting specific ERP components, combining the use of ERP components, and extracting features from the whole ERP signals, such as using the component of P2, P3, and N1/N170. For instance, Zhang, et al. [13] have proposed a temporal principal component analysis-based method for N2 and P2 components extraction in single-trial for individual subjects. It also explored the influence of the number of trials (from 10 to 42 trials), on PCA decomposition by comparing temporal correlation, and spatial correlation with conventional time-domain analysis. A stable ERP N2 component with 20 trials and a P2 component with approximately 30 trials were obtained. Wang, et al. [14] reported ERP findings for visual stimulus classification, where the stimuli include four categories: building, car, cat, and face, and 64-channel EEG equipment was used to acquire the EEG signals which were extracted for the corresponding ERP components after preprocessing the signals. The classification results for two class problems using individual ERP components with Fisher LDA (Fisher Linear Discriminant Analysis) classifier were above 50% detection accuracy, while the combined ERP components slightly enhanced the overall classification performance by 5%. The dataset of Wang et al. [14] was re-analyzed by Qin, et al. [15] and focused on EEG signals captured from the occipital lobe. Each visual stimulus data is considered independently as a subspace, and features extracted in each subspace were then fused using principal component analysis (PCA). The reported classification results based on kernel SVM was 72.57% accuracy, which is 6% higher than the results reported by Wang et al. [14]. Zhang, et al. [16] proposed a data augmentation approach for single trial detection based on a generative adversarial network to enhance the classification performance. The results proposed a 73% reduction of real subject data and acquisition cost and enhances the general classifier performance. Parashiva and Vinod [17] proposed a single-trial detection method based on temporal domain features, i.e., the standard deviation between the two categories of trials, for discriminating the correct and error trials employing a modified power-law based transformation. The reported results were presented from a sample of 10 subjects, and the average sensitivity and specificity were 86% and 92% respectively, for discriminating correct vs error trials. Similarly, Wirth, et al. [1] reported a single-trial classification method for discriminating between different error trials. The authors used two datasets were used with 25 and 14 participants to discriminate between error trials. The classification results show mean overall accuracy of 65.2% and 65.6% for two experimental tasks.

Previously we have developed feature extraction methods for spontaneous EEG recordings [18, 19], such as eyes open and eyes closed recordings, and clinical EEG recordings for detecting epileptic seizure activities, where the authors have used discrete wavelet transform (DWT) with *db*4 mother wavelet as the *db*4 wavelet is most appropriate for detecting seizure spikes. Further, in our previous work [18, 19], the arithmetic coding technique was used to convert the wavelet coefficients into bitstreams because the spontaneous EEG recordings have a relatively long duration, and the signals have the potential of high redundant information. Also, the arithmetic coding technique provides superior results in reducing the redundant information in long signals relative to short-length signals like ERPs. However, for ERPs signals, the *db*4 wavelet does not provide a good resemblance with the ERP waveforms. Thus, the previously developed features extraction method for spontaneous EEG analysis was not promising enough to detect visual events in ERPs signals. However, it has been reported from previous single-trial analysis studies that the biorthogonal B-spline wavelet is the most suitable mother wavelet for ERPs signals [20]. Moreover, the ERPs signals are time-locked short EEG segments, usually from 500ms to 2000ms long. Thus, the discrete wavelet transform with a biorthogonal B-spline wavelet could be a suitable combination to extract the ERPs from the background EEG signals more efficiently, and the Huffman coding could be a choice to use for reducing the redundancy and computing the features for ERPs.

Machine learning techniques proved helpful in brain-computer interface (BCI) applications e.g., to control motor prostheses. This kind of application requires accurate and fast detections of physical motor events corresponding with neuronal activity, which could be achievable with the use of machine learning. Many analysis methods and machine learning tools are reported to be used to achieve such accuracy in ERPs [21–23]. However, the ERP responses achieved through visual stimuli are less regular and also have a low signal-to-noise ratio as compared to the motor control task signals. Therefore, the classification of such ERPs is a challenging task and requires a robust feature extraction method that could give the best classification results by using machine learning classifiers. Machine learning classifiers such as *k*-NN and SVM have been used for the classification of mental states, diagnosis of mental disorders, and/or separation of different categories of stimuli based on EEG and ERP signals [24]. It is also reported that *k*-NN and SVM are also valuable for the detection of other health abnormalities, such as Purwar, et al. [25] reported the detection of mesangial hypercellularity MEST-C score in immunoglobulin using deep CNN. Similarly, in other studies, Purwar, et al. [26] and Purwar, et al. [27] reported the detection of microcytic hypochromic using cbc and blood film features extracted from convolution neural networks by different machine learning classifiers and using a fusion of deep image and clinical features for classification of Thalassemia patients.

The study aims to develop a feature extraction method that could efficiently extract a compact set of useful information from background EEG segments for event-related potentials (ERPs) to detect visual events from evoked potentials accurately. The proposed method for ERPs would be a cost-effective feature extraction technique that could be used in the classification of visual events. The present work chooses the DWT with the biorthogonal B-spline wavelet to decompose the EEG segments up to several levels and get the DWT coefficients. Then a thresholding technique is applied to discard unnecessary coefficients and retain only significant coefficients that hold the relevant information of the ERP signal. The retained coefficients are encoded into bitstreams with Huffman coding to extract features. Moreover, the proposed feature extraction method can assist in the real-time detection of visual event-related potentials, particularly desirable in applications of brain-machine interfaces or brain-computer interfaces.

The organization of subsequent sections of the paper follows: the section Materials and Methods provides details of the experimental task, participants’ information, experimental procedure, EEG recording & preprocessing, details steps of the proposed feature extraction method, and analysis; the section’ Experimental Results and Discussion’ presents the findings of the study, comparison results, and provides discussion on the study findings and relevant to previous studies, and reporting the limitations of the study for future research; finally the paper is concluded.

## Materials and methods

This section provides the detail of data collection (including participants’ information, visual object detection task, experiment procedure, and preprocessing steps) wavelet transform, an explanation of the proposed method, and an overview of machine learning classifiers used in this work.

### Participants

The sample size in this experiment was sixty-eight ostensibly healthy participants recruited for participation. Their age range was between 18-to-30 years and the mean (M) and standard deviation (SD) were 23.66, and $\pm 3.63$, respectively. All of them had normal or ‘corrected to normal’ vision and were free from neurological disorders, medication, and hearing impairments. They signed a written informed consent document before starting the experiment as per the defined study protocol. This analysis is part of a research study that was approved by the Human Research Ethics Committee of the collaborating institution [28].

### Visual object detection task and stimulus

**Fig. 1:**
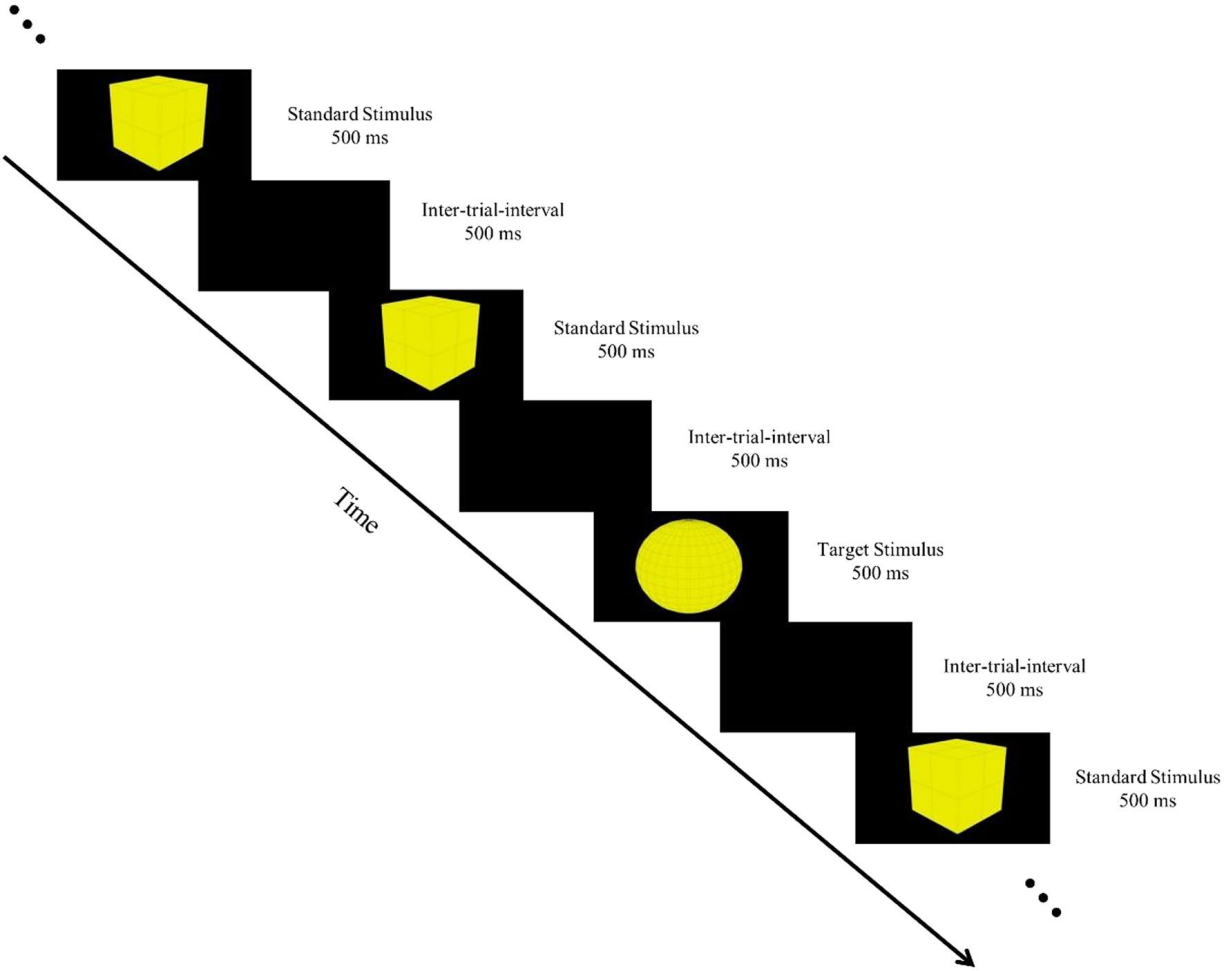
An illustration of the visual object detection task

The visual object detection task is used for studying visual evoked potentials in ERP studies. The presentation of visual stimuli allows us to examine the neural activity elicited during attention-demanding cognitive events [29]. In the experimentation, the visual object detection task was performed by all the participants, where two shapes: a box and a sphere, were used to represent the target and standard stimuli. The duration of every trial was 1000ms in which the visual stimuli, either standard or target, were presented for 500ms time and 500ms time was used between two consecutive events as an inter-trial-interval (ITI), see Fig. 1. The task demands the participants to use the button ‘0’ from the numeric keyboard to record responses against a target stimulus, and not to respond when a standard stimulus appears. All the participants were instructed to avoid errors and respond quickly as possible. Thus, the presentation software captured the reaction time and correct responses for target stimulus detection synchronized with the neural activities. In the task, a total of one hundred and thirty-five trials were used, in which thirty percent of trials possessed target stimulus trials and seventy percent possessed standard stimulus trials. Accordingly, forty trials belong to target stimulus events and ninety-five trials belong to standard stimulus events. The task was approximately four minutes long, which was adopted with modification from a previous ERP [30].

### Experiment procedure

The schedule of the data collection was communicated to all the participants, and according to their availability, experiments were conducted individually at the specified time. When a participant arrived, he/she was informed about the experimental procedure and instructed according to the defined study protocol. Then the participant was seated in a partially sound-attenuated EEG room for preparation. Head measurement was taken and accordingly an EEG electrodes cap of appropriate size was set up as per the device manual, and thus the participants completed the experiment, including the visual object detection task, which was completed in around four minutes. The experiment was run on a screen attached to a laptop to synchronize the stimuli presentation with the EEG recording using E-Prime Software [31].

### EEG recording

The EEG signals were captured during the experimental task from all the participants over the whole scalp of 128 locations by using the EEG device HydroCel Geodesic Sensor NetAmps 300 from Electrical Geodesic Inc., Eugene, OR, USA. In the EEG data acquisition, the EEG sensors were referenced to the Cz location, and raw EEG signals were amplified with the NetApms300 amplifier of EGI in net-station software. The filtering setting was kept as bandpass 0.1 to 100 Hz and a notch filter of 50 Hz; while the impedance was set below 50 K$\Omega$ ensuring a good signal-to-noise ratio as per the manufacturer’s guidelines [11]. The continuous EEG signals were digitized with a sampling rate of 250 Hz.

### EEG pre-processing

**Fig. 2:**
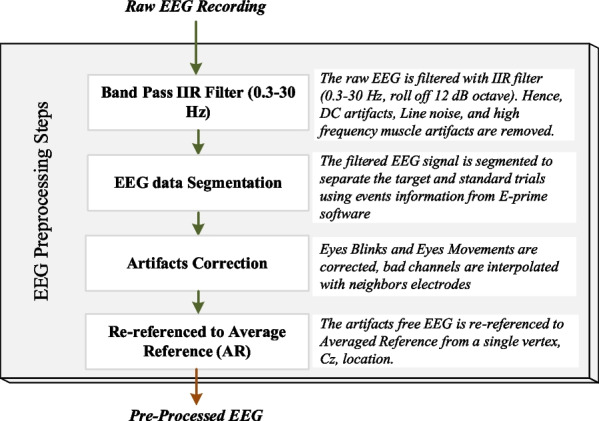
EEG preprocessing for ERPs analysis

EEG is a non-stationary and highly time-varying sensitive signal. During acquisition, EEGs are highly vulnerable to the external environment. The vulnerability to the external environment allows different unwanted interferences to the EEG, known as artifacts. The causes of artifacts could be due to physical activities, such as cardiac activity (electrocardiogram, ECG), muscle contraction (electromyogram, EMG), ocular activity caused by eye movement and/or blinking (electromyogram, EOG), and interference from the EEG device (DC drift) itself and the line (line noise at 50Hz). These artifacts mixed with the EEG activity can greatly mislead the analysis results, especially in time-locked EEG such as ERPs, where EEG is synchronized with the visual or audio stimuli. Therefore, the EEG signals need proper pre-processing before considering the signals for extracting events relevant information, i.e., feature extraction, for further analysis. In general, the artifacts due to the EEG device itself and the line noise can be removed from the recordings by using band-pass filtering and notch filter. The artifacts due to physical activities can be handled with Blind Source Separation [32], spatial filtering, and adaptive filtering. In this study, the data pre-processing of raw EEG recordings was carried out with the following steps. The pre-processing included the EEG segmentation, as shown in Fig. 2.

1. a bandpass filter 0.3–30 Hz, roll-off 12 dB octave was applied and discarded high-frequency artifacts and DC components.
2. the EEG signals were segmented with 600ms length including 100ms pre-stimulus time as a baseline and 500ms post-stimulus time for obtaining the individual EEG trials.
3. individual EEG segments (trials) if contaminated with eye blinks and eye movements, i.e., if the amplitude exceeded $\pm 90\mu$V, were corrected with the independent component analysis (ICA) method [32].
4. The EEG segments (trials) were visualized to detect the electrodes that had lost contact in the event of widespread drift, as well as bad channels in the segments, which were corrected with the spherical spline method [11].
5. Finally, the data were re-referenced to the averaged reference from a single vertex Cz electrode and subsequently exported into *.mat format for further analysis as pre-processed EEG signals. The timestamps of stimulus onset, response, and offset were extracted from the stimuli presentation software.

### Wavelet transform

The wavelet transform is the inner product of a given signal *x*(*t*) with dilated and translated versions of the wavelet function $\Psi _{a,b}(t)$ is defined as:

1 $$\begin{aligned}{} & {} \begin{aligned} W_\Psi X(a,b)=\langle x,\Psi _a,_b\rangle \end{aligned} \end{aligned}$$

2 $$\begin{aligned}{} & {} \begin{aligned} \Psi _a,_b=\mid a\mid ^{-1/2}\Psi (\frac{t-b}{a}) \end{aligned} \end{aligned}$$

The parameters $a,b\in \Re$ represent the scale and translation, respectively. The scaling parameter dilates or compresses the wavelet function and the translation parameter changes its location [33]. The correlation of the signal *x*(*t*) with the dilated/contracted versions of the wavelet function $\Psi _{(a,b)} (t)$ provides the low/high frequency components. Practically, the wavelet transform is defined at discrete scales $a_j=2^j$ and times $b_{(j,k)}=2^j k$, called discrete wavelet transform (DWT). The DWT successfully divides the given signal into approximations and details coefficients at different scales. The lower scales give information about high-frequency components and the high scales give information about low-frequency components. The DWT decomposition in the present study is given in the description of the proposed method section.

### Huffman coding

Huffman coding is a widely used technique for eliminating data redundancy and generating optimal codewords for data compression. For a given set of alphabets or data symbols with their probabilities of occurrences, it produces a set of variable-length codewords, having the shortest average length, and allocates the codewords to the given data symbols. First, it creates a sequence of source reductions by looking at the frequencies of occurrence (the probabilities) of the source symbols and taking the bottommost probabilities symbols into a single symbol that substitutes the source reduction. Repeating this process till reducing the source to two symbols. Second, to encode each reduced source, starting smallest source and going back to the original source. The minimal length binary code (0 and 1) is assigned to two symbols in an arbitrary style. Repeating this process for each reduced source symbol till it reached the original source. The average length of the final code can be obtained as:

3 $$\begin{aligned} \begin{aligned} {L}_{avg}=\Sigma _{(i=1)}^{(J^n)} P(a_i )l(a_i ) \end{aligned} \end{aligned}$$

Where $\textit{P}(a_i)$ represents the probability, and $\textit{l}(a_i)$ denotes the code length of the $i^{th}$ symbol. Huffman coding is useful in many applications, such as data compression. In the present study, the Huffman coding method is used as one step after discarding wavelet coefficients with a threshold in the EEG feature extraction for single-trial analysis. Thus, the Huffman coding can transform the wavelet coefficients into bitstream which would be a compact representation of the ERPs.

### Proposed Feature Extraction Method for ERPs Analysis

**Fig. 3:**
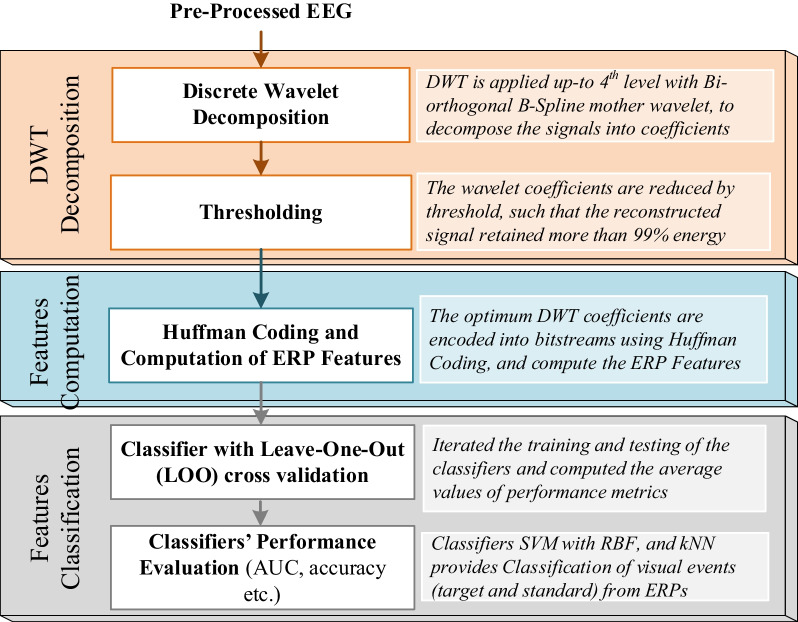
Illustration of the proposed method for single-trial analysis and classification of visual events

The proposed method as illustrated in Fig. 3 consists of three main steps, including (i) DWT decomposition, (ii) Features Computation, and (iii) Features Classification, besides the pre-processing steps which are described in the previous section ‘EEG Pre-processing’.

**Fig. 4:**
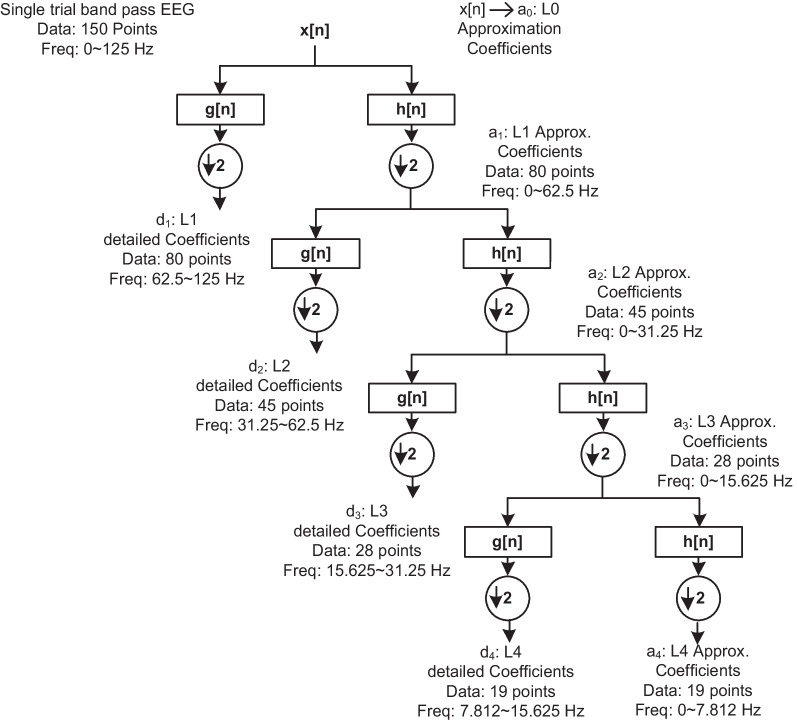
Fourth-level DWT Decomposition, where d1 refers to detailed coefficients and a1 denotes the approximation coefficients at level 1, L refers to level, Freq. stands for frequency in hertz

**Fig. 5:**
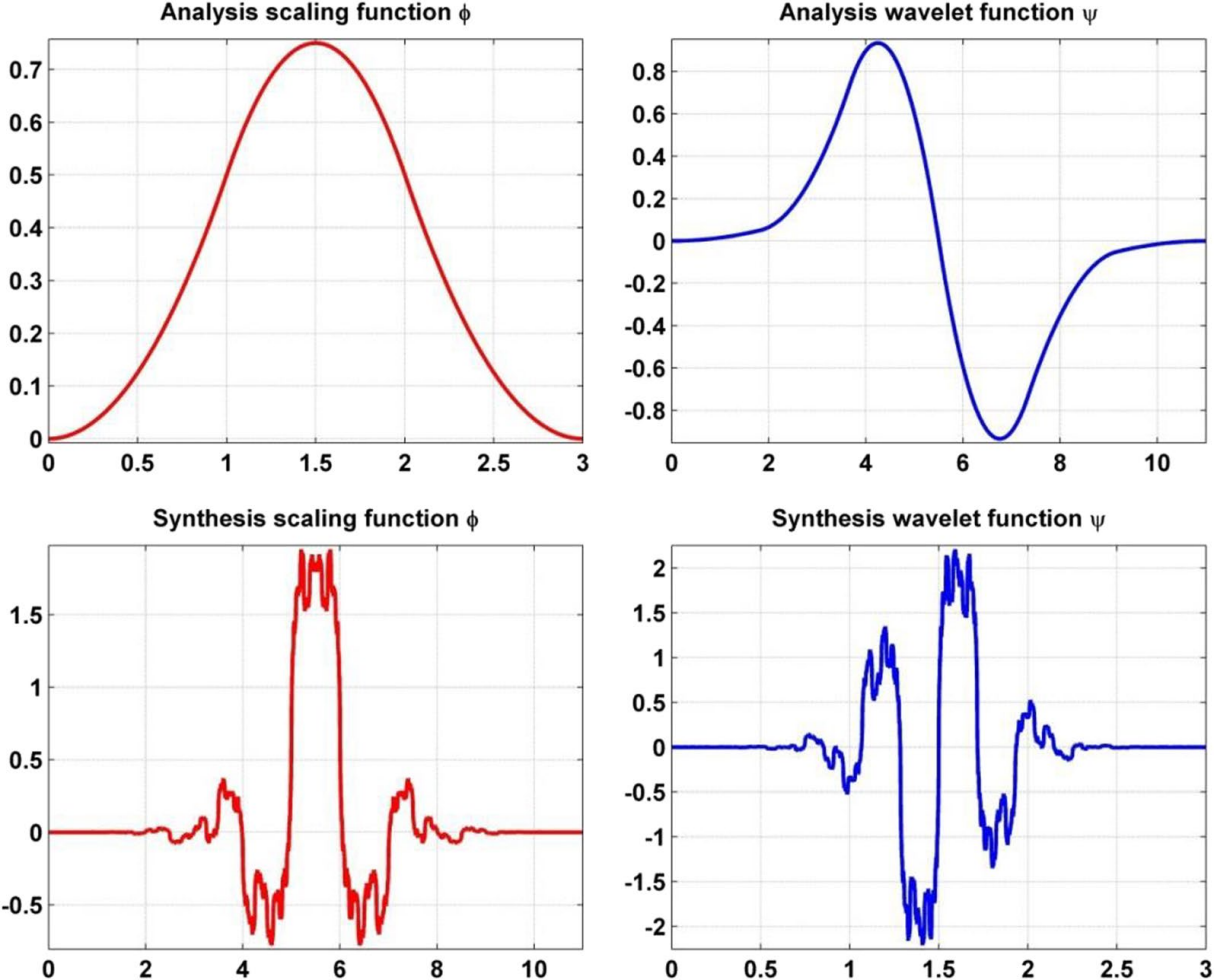
Bi-orthogonal B-Spline mother wavelet (bior3.5) analysis and synthesis

The pre-processed EEG signal, represented as *x*[*n*] is decomposed by applying the DWT up to the fourth level with a biorthogonal B-spline wavelet with three vanishing moments in the reconstruction (synthesis) wavelet and five vanishing moments in the decomposition (analysis) wavelet, produces approximation and detailed coefficients, see Fig. 4. The bi-orthogonal B-Spline was selected as the basic wavelet function due to its reported suitability for the analysis of ERPs data [20, 34, 35]. B-splines have compact support and possess a shape (see Fig. 5) that resembles the ERP waveform [20], providing an optimal resolution in time-frequency for the input signal, which implies that the evoked responses are localized in a few coefficients. The localization of evoked responses in a compact number of wavelet coefficients allows the representation of the ERP signal within a few optimal coefficients and discards non-significant coefficients while fulfilling certain signal quality criteria, i.e., 99% energy in a reconstructed signal. The procedure of DWT in the decomposition applies consecutive lowpass *h*(*n*) and high pass *g*(*n*) filters, where the high pass filter *g*(*n*) represents the discrete mother wavelet and the low pass filter *h*(*n*) represents its mirror version [36]. The *h*(*n*) and *g*(*n*) filters have a cutoff frequency that is one-fourth of the input EEG signal’s sampling frequency. At the start of decomposition in the first level, the lowpass *h*(*n*) and high pass *g*(*n*) filters are concurrently applied to the input EEG signal and produce the corresponding outputs, which are referred to as approximation coefficients or $(A_1)$ and detailed coefficients or $(D_1)$, respectively. The approximation and detailed coefficients are the dot product of the EEG signal and the specified basis function. Mathematically, in the $i^{th}$ level of decomposition the approximation coefficients $A_i$ and the detailed coefficients $D_i$ can be expressed as below:

4 $$\begin{aligned} \begin{aligned} A_i=\frac{1}{\sqrt{M}} \Sigma _{n} X(n).\varphi _{j,k}(n) \end{aligned} \end{aligned}$$

where, $\varphi _{j,k}(n)=2^{-j/2}h(2^{-j}n-k)$ is the scaling function.

5 $$\begin{aligned} \begin{aligned} D_i=\frac{1}{\sqrt{M}} \Sigma _{n} X(n).\phi _{j,k}(n) \end{aligned} \end{aligned}$$

where, $\phi _{j,k}(n)=2^{-j/2}h(2^{-j}n-k)$ is the wavelet function.

Here the parameters used in the above two equations represent the DWT decomposition which is used up to level 4, so $J=4$; while the length of the discrete EEG signal *x*[*n*] is denoted with *M*. Likewise, the value of $n= 0,1,2,...,M-1$; and the value of $j=0,1,2,...,J-1$; and value of $k=0,1,2,...,2^{j}-1$.

The outputs of DWT decomposition including the last approximation $(A_i=4)$ and all the detailed coefficients $(D_{i=1,2,3,and 4})$ are denoted with $D_{jk}$ and diminished after using a certain threshold value $\alpha$ which discarded the non-significant coefficients.

6 $$\begin{aligned} \begin{aligned} \widehat{D}_{jk}= {\left\{ \begin{array}{ll} D_{jk}, &{} \text{ if } \mid D_{jk}\mid \ge \alpha , \\ 0, &{} \text{ if } \mid D_{jk}\mid <\alpha , \end{array}\right. } \end{aligned} \end{aligned}$$

The reconstructed signal is ensured to have more than 99% energy after using the threshold value $\alpha$.

7 $$\begin{aligned} \begin{aligned} Energy (E)=\frac{(100\times \parallel X_r\parallel _{2}^2)}{\parallel X\parallel _{2}^2}>99\% \end{aligned} \end{aligned}$$

Here the variables $X_r$ represents the restored EEG signal and *X* refers to the original EEG signal.

The criteria reported for computation of threshold parameter $(\alpha )$ by Donoho and Johnstone [37] is used, where the standard deviation of the noise (unwanted signal) is estimated by considering the last level of the detailed coefficients vector. Moreover, hard thresholding is used and the threshold value $(\alpha )$ is expressed as follows:

8 $$\begin{aligned} \begin{aligned} \alpha ={\widehat{\sigma }}\sqrt{2logN} \end{aligned} \end{aligned}$$

Here, the number of wavelet coefficients that exist in the last level of detailed $(D_4)$ is indicated with *N*. The value of ${\widehat{\sigma }}$ is computed which is based on the median absolute deviation and expressed here as,

9 $$\begin{aligned} \begin{aligned} {\widehat{\sigma }}=\frac{median|\widehat{D}_{jk}|}{0.6745} \end{aligned} \end{aligned}$$

Here, in the above equation, the term in the denominator reflects the scale factor, depending on the distribution of $\widehat{D}_{jk}$, for normally distributed data the value is equal to 0.6745; while $\widehat{D}_{jk}$ represents the wavelet coefficients in the last level of the detailed coefficients.

Accordingly, the use of a threshold parameter ensured the quality of the reconstructed EEG signal, which is obtained after discarding the non-significant coefficients. The thresholded DWT coefficients, denoted with $\widehat{D}_{jk}$, are rounded off to the nearest integer, represented as ${\overline{D}}_{jk}$. The rounded-off DWT coefficients ${\overline{D}}_{jk}$ are converted into bitstreams by using the Huffman coding technique.

In Huffman coding, the whole sequence of DWT coefficients ${\overline{D}}_{jk}$ is assigned a codeword. The Huffman codewords represent the single-trial waveform in a compressed form. The size of DWT coefficients is reduced, resulting in a compressed single trial of the EEG signal. Accordingly, the DWT coefficients’ size is compacted, and subsequently, the signal is compressed. Finally, the Huffman coding output bitstreams provide the computation of features denoted with *F* as follows:

10 $$\begin{aligned} \begin{aligned} F=\frac{1}{CR}\times 100 \end{aligned} \end{aligned}$$

Where,

11 $$\begin{aligned} \begin{aligned} CR=\frac{(x)}{(x_c)} \end{aligned} \end{aligned}$$

Here, (*x*) represents the size of orignal ERP signal, and $(x_c)$ reflects the size of compressed ERP signal. This process is iterated for each participant’s data, including all the channels, visual events (target and standard), and trials. The feature matrix *F* for a single subject for each visual event can be represented as:

12 $$\begin{aligned} \begin{aligned} F= \begin{bmatrix} 1 &{} \cdots &{} n \\ \vdots &{} \ddots &{} \vdots \\ m &{} \cdots &{} m\times n \end{bmatrix} \end{aligned} \end{aligned}$$

where, *m* denotes the number of trials for the target or standard event and *n* represents the number of channels.

**Fig. 6:**
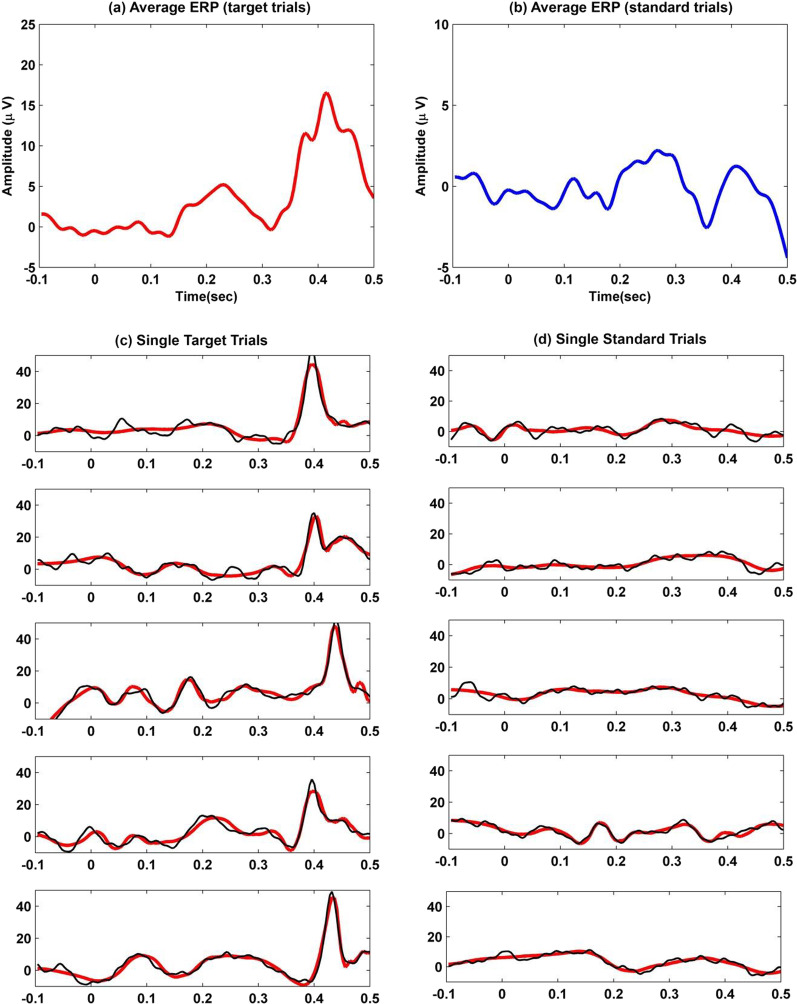
Average ERP from 40 trials of one subject at Pz **a** target trials and **b** standard trials. Single trials bandpass EEG signal (black), reconstructed ERP signal from optimum wavelet coefficients (red) of one subject (five out of 40 trials) at Pz **c** target trials, and **d** standard trials

The literature reported the wavelet decomposition for various applications of EEG classification with different levels, for instance, level 3, level 4, or higher [19, 38]. It is further reported that the range of basic EEG rhythms, including delta, theta, alpha, beta, and gamma waves, correspond to DWT coefficients located in $D_1$ to $D_4$ and $A_4$ [38]. Thus, level 4 is chosen for DWT decomposition because of the corresponding frequencies in the EEG signals collected in this study. Moreover, it was observed that the number of wavelet coefficients that can be discarded without disturbing the quality of EEG signals was high in decomposition level 4, which allowed only thirty percent of the wavelet coefficients for the reconstruction of the original signal while retaining 99% of the signal energy. However, examining higher than four levels of DWT decomposition did not produce a significant rise in the number of discarded wavelet coefficients, see, Fig. 6 shows an average ERP from 40 trials of one subject at *Pz* location for target trials and standard trials. Also, displays five trials for a single trial bandpass EEG signal (black), and the superimposed reconstructed ERP signal from optimum wavelet coefficients (red) of one subject at *Pz* both for target and standard trials.

### Machine learning classifiers and cross-validation

Machine learning classifiers are functions taking input data as independent variables and making predictions about the input data corresponding to the relevant class, the data points belong to [39]. To determine the efficiency of the proposed method in ERP single trials classification, we have used two machine learning classifiers: *k*-nearest neighbors (*k*-NN) with $k=5$; and Support Vector Machine (SVM) with radial basis function (RBF). Both classifiers are supervised learning techniques. The *k*-NN works to find a testing sample’s class by the majority class of the *k* nearest training samples, that is, a class label is allocated to the new instance that is the most common class amongst the *k* nearest neighbors in the feature space, see [39] for details of *k*-NN and machine learning classifier. The SVM is based on large margin theory and uses the decision function with maximum margin. The kernel trick of SVM allows the successful handling of non-linear separable data. The RBF provides a good classification performance in many applications including EEG data, and it is a well-known SVM kernel function. There are two parameters for SVM classifier with RBF kernel, the first one is the soft margin parameter C and second one is the RBF kernel parameter ($\gamma$), both were tuned with utilizing a *k*-fold cross-validation strategy.

A standard procedure for assessing the performance of a classifier on a given dataset is the *k*-fold cross-validation (CV) method [40]. This method randomly splits the dataset into *k* disjoint folds with identical sizes, where $k-1$ folds are used as training data, and one-fold is retained as validation for testing the machine learning model. The CV process is then iterated k times with each of the *k*-folds used exactly once as the validation data. Thus, the model produces *k* times the performance metrics. Accordingly, the performance of the classifiers can be evaluated by taking an average over *k* values of the metrics, such as accuracy, resulting from the *k*-fold cross-validation. The CV approach has the advantage of using all the data points (observations or instances) in the given dataset for training and testing and each observation is utilized exactly once for validation. Further, as the value of *k* is increased, the variance of the resulting estimate could be reduced. The computation cost for running the training algorithm for *k* times might be high if the dataset is significantly large; in that case, it can be handled by selecting a reasonable *k* value, for instance, 10-fold. In the present study, leave-one-out (LOO) cross-validation is adopted as the dataset is relatively small. A more description of the LOO is provided in the experimental setup and results section.

## Experimental setup, classification findings, and discussion

The experimental setup employed for extracting ERP features and classification of visual stimuli to assess the proposed approach for ERPs analysis is explained in this section. We start with feature extraction and then train/test the machine learning classifiers and cross-validation, reported the classification results, compared the results of the proposed approach for ERP extraction with state-of-the-art methods, and finally, the findings are discussed.

### Experimental setup

For each subject, 40 sets of ERPs have been extracted, i.e., responses to target and standard stimuli recorded over the scalp. Usually, ERPs are analyzed on the midline electrodes, such as *Fz*, *Cz*, and *Pz*. However, in this study, a dense array EEG of 128 electrodes data was used to study the brain responses all over the scalp during the given visual events. Each single-trial ERP was decomposed into four levels of DWT detail and approximation coefficients using a bi-orthogonal B-Spline wavelet. The wavelet coefficients correspond to different frequency bands, see Fig. 4. All the detailed and last approximation coefficients were considered for analysis because the input EEG signal to DWT decomposition was band passed $0.3\sim 30 Hz$. The number of coefficients extracted at each level depends on the input signal length and the analysis wavelet (the number of its filter tabs [34]). Each single-trial recording was selected from the continuous EEG 100ms before the stimulus onset and lasted for 500ms after the stimulus onset. The extracted coefficients corresponded to -100ms to 500ms, i.e., 600ms duration of every single trial. The optimum DWT coefficients retained the relevant information of evoked responses for each trial. Since the amplitude of the wavelet coefficients is correlated with the ERPs [20]. Thus, the variations in the standard and target trials can be detected by computing the ERP features from the joint use of DWT coefficients with Huffman coding. The Huffman coding further reduced the size of DWT coefficients, reducing the redundancy in the computation of features.

After the feature extraction, machine learning classifiers are applied to detect the trials corresponding to class 1 and class 2 or discriminate as standard and target trials. To improve both the strength of the classifier and its ability to generalize to new data, the implementation of the classifier is cross-validated with leave-one-out (LOO). The leave-one-out (LOO) is *k*-fold cross-validation in which the *k* is equal to *N* (where *N* is the number of instances or data points in the dataset). Thus, the number of folds is equal to the number of data observations. Accordingly, the classifier is trained and tested k times iteratively. Each training on $k-1$ of *k* folds and the testing is done on the remaining one fold (unseen data) [39]. In this process, each trial is used at least one time as test data. The classification results are then observed from the accuracy of testing the respective unseen $k^{th}$ fold, and the accuracy, sensitivity, specificity, and precision, are averaged across the *k* folds [39].

The machine learning classifiers SVM and *k*-NN are assessed for the classification performance on ERPs visual stimuli. For this purpose, different model evaluation metrics are used such as sensitivity (*SEN*), specificity (*SPC*), accuracy (*ACC*), precision (*PRC*), and *ROC* analysis. The *SEN*, *SPC*, *ACC*, and *PRC* are described as follows.

13 $$\begin{aligned}{} & {} \begin{aligned} SEN=\frac{TP}{TP+FN} \times 100\% \end{aligned} \end{aligned}$$

14 $$\begin{aligned}{} & {} \begin{aligned} SPC=\frac{TN}{(TN+FP)} \times 100\% \end{aligned} \end{aligned}$$

15 $$\begin{aligned}{} & {} \begin{aligned} ACC=\frac{(TP+TN)}{(Total Cases)} \times 100\% \end{aligned} \end{aligned}$$

16 $$\begin{aligned}{} & {} \begin{aligned} PRC=\frac{(TP)}{(TP+FP)} \times 100\% \end{aligned} \end{aligned}$$

The number of trials correctly identified corresponding to ‘class 1’ by the system is represented as ‘true positive or *TP*’; the number of trials correctly identified corresponding to ‘class 2’ by the system is referred to as ‘true negative or *TN*’; the number of incorrectly identified trials as ‘class 1’ by the system are denoted as ‘false positive or *FP*’ and the number of incorrectly identified trials by the system is named as ‘false negative or *FN*’.

### Classification findings

The proposed feature extraction method for the classification of visual stimuli based on single-trial ERPs presents promising classification results for detecting visual events, i.e., standard events, or target events. The extracted features from single trials of all the participants were checked for potential outliers in the data. A standard statistical procedure was followed for detecting outliers in the extracted features, i.e., the three-sigma rule, which states that any feature would be considered an outlier if the feature value is greater than +3 standard deviations from the mean value, or less than -3 standard deviations from the mean value [41]. The features were visualized through box plots. In addition, a normality test was applied to the extracted features to confirm that the features meet the normality criteria defined by the Shapiro-Wilk test [42]. Overall, none of the participant’s data met the outlier criteria to be excluded. However, some features deviated from the mean value in a few participants’ data associated with the electrodes placed on the most external row of the EEG 128 electrodes cap, such as electrodes placed near the neck and/or near the face. Consequently, the deviated features were corrected using the data imputation method, which replaced the deviated feature’s value by considering the mean of the features extracted from the neighboring electrodes. Thus, all the sample was included in the classification results. Since the objective of the study is to detect the visual events through single-trial ERPs, therefore, individual participant data was used for classification, and the overall mean and standard deviations of the classification results across participants of both the classifiers, *k*-NN and SVM, are depicted in Table 1, which shows promising classification results for Standard and Target visual events. Both SVM and *k*-NN classifiers achieved promising results in detecting visual events. However, the results of the SVM classifier are relatively superior to the *k*-NN. The SVM classifier achieved an average accuracy of 93.60% compared to the 90.80% accuracy of the *k*-NN classifier across the participants.

**Table 1:** Classification of target and standard events using a Support Vector Machine (SVM) with RBF kernel and k-nearest neighbor (*k*-NN) classifier with the *k*= 5

	Accuracy	Sensitivity	Specificity	Precision	AUC
SVM	93.60 ± 6.5	93.55 ± 4.5	94.85 ± 4.2	92.50 ± 5.5	0.93 ± 0.3
*k*-NN	90.80 ± 7.4	91.50 ± 6.2	90.20 ± 7.3	91.45 ± 6.5	0.91 ± 0.5

Data is arranged as mean±standard deviation, AUC stands for ‘Area Under the ROC Curve’. RBF stands for ‘radial basis function’

**Fig. 7:**
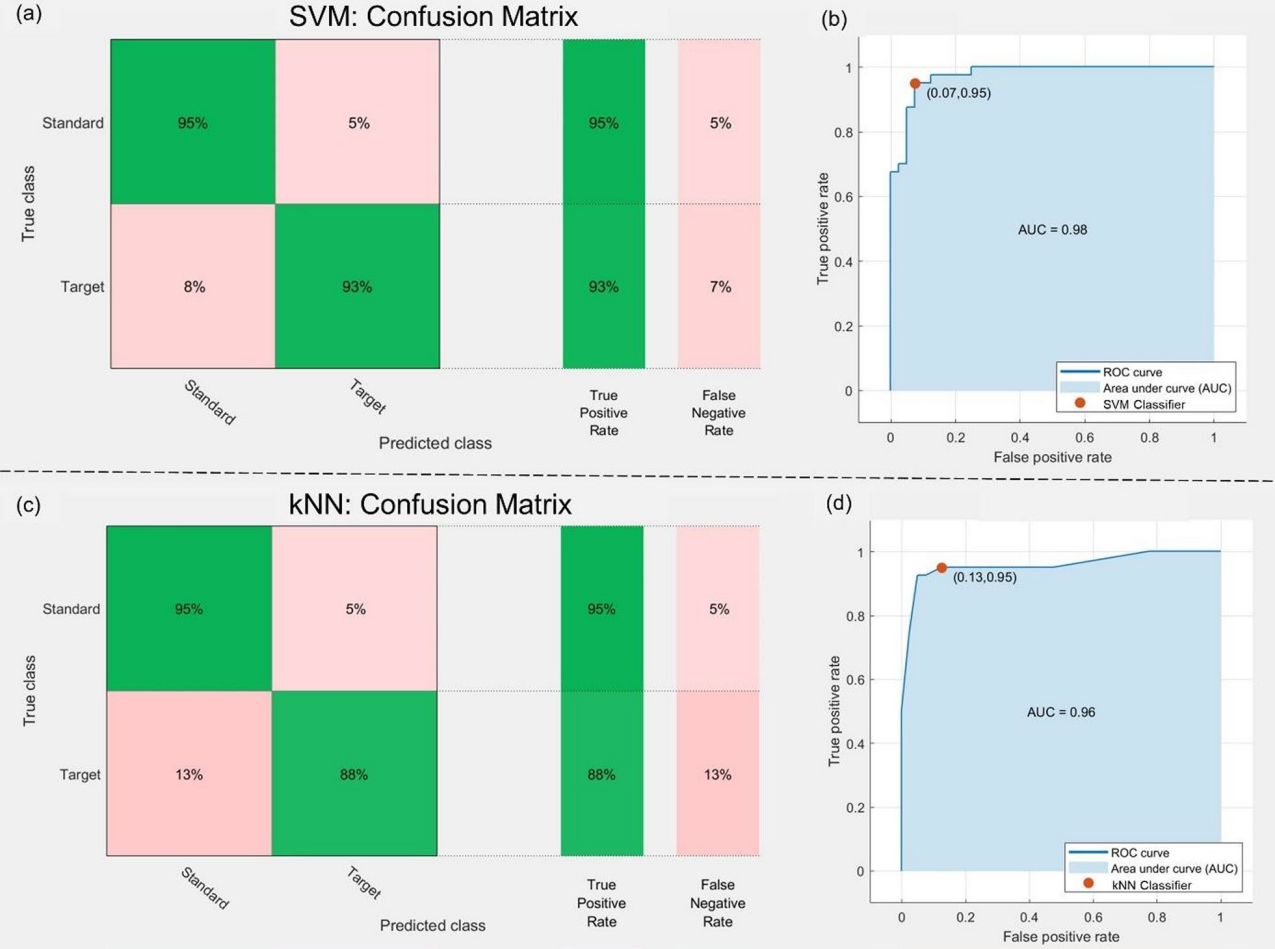
Illustration of **a** Confusion Matrix and **b** Area under the ROC Curve of SVM classifier, **c** Confusion Matrix and **d** Area under the ROC Curve of *k*-NN Classifier

**Fig. 8:**
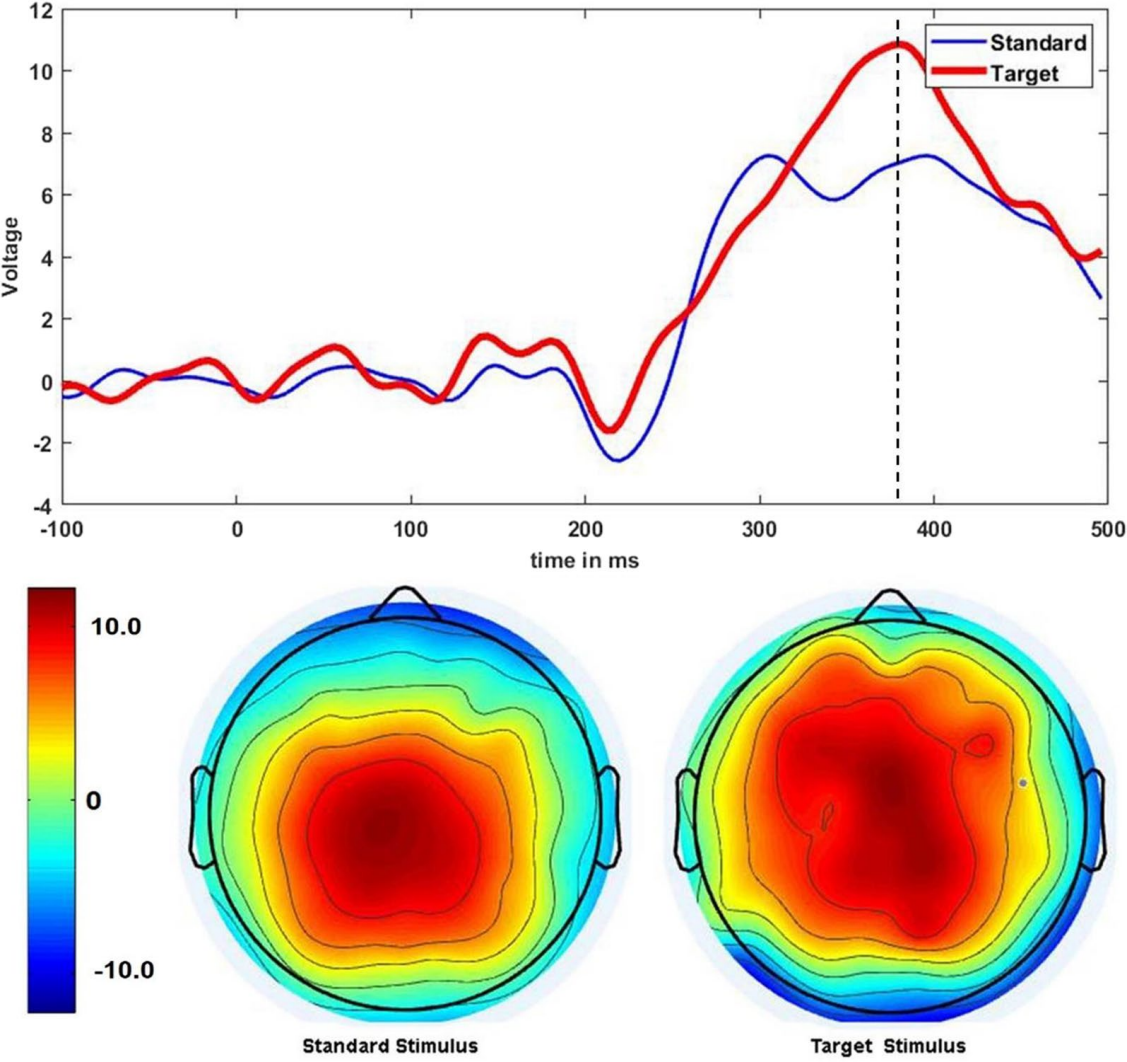
An illustration of averaged ERPs over multiple trials of a single subject for the Standard and Target events elicited at the Pz site with corresponding whole-brain topographic maps at peak amplitude, shown as a vertical black dotted line

In Fig. 7, the confusion matrix, ROC (receiver operating characteristics) curve, and Area Under the ROC Curve (AUC) for both SVM and *k*-NN classifiers are shown, produced from one participant’s data for illustration. The ROC curve demonstrates the true positive rate (TPR) on the $y-axis$, and the false positive rate (FPR) on the $x-axis$, reflecting the performances of the SVM and *k*-NN models. The AUC generally represents the degree of separability of a model, accordingly, a higher AUC value for SVM indicates a superior classification between standard events and target events relative to *k*-NN. The neural activities evoked by the target and standard stimuli are closely resembled except after a 300ms time window, see Fig. 8, where a strong P300 component appears in the target trials, and the corresponding amplitude of the standard trials is relatively low. For illustration, an averaged ERP of a single participant for the Standard and Target events elicited at the *Pz* site is visualized, and the whole scalp topography, covering all the electrodes at the peak amplitude, shown as the dotted line in Fig. 8, demonstrating the differences between the standard and target evoked responses.

### Comparison of proposed approach with existing methods

The proposed single-trial ERP extraction method is compared with the relevant previous studies by implementing their reported methods on the dataset used in this study. Two recent relevant studies on single-trial extraction were implemented; the first one was reported by Zhang, et al. [13], which focused on factor analysis with the use of principal component analysis (PCA) for single-trial ERP extraction. The single-trial ERP features extracted were tested/validated with both the SVM and *k*-NN classifiers. The obtained results were quite low than the results of the proposed method (see, Table 2). Similarly, the implementation of the second previous study here was reported by Lee, et al. [43], which focused on the use of one-unit independent component analysis (ICA) with reference. The single-trial ERP features extracted with the ICA-based method were tested/validated with both the SVM and *k*-NN classifiers and found below the achieved performance of the proposed method (see, Table 2). Thus, the proposed single-trial ERP extraction method outperformed the state-of-the-art methods in extracting single-trial ERPs and the classification of visual events. Finally, the comparison with previous relevant studies confirmed the robustness of the proposed single-trial ERP extraction and classification of visual events with machine learning.

**Table 2:** Comparisons of findings (extraction of ERP and classifiers accuracy) with previous studies for single-trial ERPs Extraction

	PCA-based Method[13]	ICA-based Method[43]	Proposed Method
SVM	74.30 ± 7.3	77.30 ± 6.8	93.60 ± 6.5
*k*-NN	71.70 ± 6.6	72.70 ± 5.9	90.80 ± 7.4

Data is arranged as mean±standard deviation

## Discussion

The conventional analysis of event-related potentials is based on the study of the average responses to differentiate the categories of events, such as target and non-target. The averaging method needs a series of repeated multiple trials of EEG to improve the signal-to-noise ratio and detect the ERPs. In addition, the ensemble averaging discarded critical information about processes developing during an experiment, such as sensitization and habituation [20]. On the contrary, single-trial analysis can detect ERPs in a single or very few trials, rather than requiring an average of repeated multiple trials. However, the detection of ERPs in the single-trial analysis is a challenging task due to their low amplitude compared to spontaneous EEG activity. ERP components, including P300, can be varied systematically or unsystematically across the trials. The decrease in amplitude of the ERPs due to habituation is an example of systematic changes in neural response [44]. Previously, it is reported that P300 amplitude is decreased with repeated presentation of the target stimulus without any strong shifts in latencies [45]. In addition, it is also shown in an animal study that exponential amplitude decays of ERPs elicited with auditory clicks, while in the early trials, a significant increase in the amplitude is observed due to sensitization [3]. The neural changes can be unsystematic in the single-trial ERPs as reflected in trial-to-trial amplitude and latency variabilities (known as amplitude and latency jitters). The trial-to-trial latency variability of ERPs causes two consequences [46]: First, it blurs the average ERP waveforms and may induce or attenuate existing condition effects in amplitude and amplitude differences between conditions. Second, different extents of latency jitter across conditions may imitate ERP amplitude effects. For example, suppose two conditions have identical amplitudes but differ in variabilities of single-trial latencies. In that case, the average ERP will result in amplitude differences across conditions, which might be statistically significant. The amplitude jitter and latency jitter can be handled by using selective averages [47] and latency corrected averages [48], respectively, but their solutions still have limitations, as reported by [49].

Numerous studies have been reported on single-trial ERPs detection using the wavelet transform [20, 35, 50–52]. However, these studies have limitations, such as Quian Quiroga [51]; Quian Quiroga and Garcia [35] which previously proposed a useful wavelet-based denoising method to improve the visualization of single-trial ERPs. However, the method required prior knowledge of the time and frequency ranges in which the single-trial ERPs are likely to reside. In addition, extensive work of an expert user is also required to choose the wavelet coefficients manually [3]. In the present study, we presented a wavelet-based single-trial extraction method from the spontaneous EEG noisy background collected in a two-stimuli object detection paradigm and then utilized machine learning to classify the visual evoked responses elicited by the target and standard stimuli. We used a Bi-orthogonal B-Spline mother wavelet because of its compact support, and close resemblances with the evoked neural responses [20], resulting in a good localization of the evoked responses in the wavelet domain with a few wavelet coefficients. Thus, allowing to the representation of the single trial in a compact form. The target and standard trials elicited closely related neural responses, which raises concerns over the single-trials classification algorithms, especially at the feature extraction level. The similarity between the corresponding features of target and standard trials can make it hard for the classification algorithm to detect the given classes with high accuracy. The differences between target and standard trials, especially in P300 detection, occur after 300ms to the stimulus onset, which possibly resides in the low-frequency components. The threshold criteria used in this study mostly eliminated the high-frequency components and retained the low-frequency components, resulting in a different number of percentage optimum wavelet coefficients selected to represent the target and standard trials, achieving an acceptable level of classification results.

The novelty of this study is the joint use of discrete wavelet transforms (DWT) with Huffman coding to extract robust ERP features in the proposed method. The discrete wavelet transform with a Bi-orthogonal B-Spline mother wavelet is previously reported as the most suitable wavelet for ERPs analysis [20, 34, 35], providing an optimal time-frequency resolution implies that the evoked responses are localized in a few wavelet coefficients. Thus, the compact number of wavelet coefficients allows for the representation of the ERP signal with few optimal coefficients, which were further compressed with Huffman coding to calculate the ERP features finally and subsequently classify them. With Huffman coding, DWT allows locating signal compression, the limit to which a signal can be compressed, which is an important property of EEG signals that varies with the brain’s states. For instance, a resting-state EEG signal would have a high probability of compression relative to an EEG signal recorded during a cognitive task. Furthermore, the filter bank implementation of the DWT takes *O*(*N*) time. The encoder and decoder of the Huffman coding technique are also in linear time [53]. Thus, the time complexity of the proposed method is linear, i.e., *O*(*N*).

The single-trial ERP extraction method reported by Zhang, et al. [13] focused on factor analysis with the use of principal component analysis (PCA) for single-trial ERP extraction. PCA is a linear transformation method that aims to identify the underlying components of the EEG signal that capture the most variance in the signal. In the context of ERP analysis, PCA can identify the components of the signals that are related to specific ERP component, like P300 or N100. The benefit of using PCA could be the speed, which makes it ideal for large EEG data. However, the limitation of PCA for ERP extraction is that the components may correspond to stimulus-relevant information such as desired ERP components, and stimulus-irrelevant information such as artifacts. Similarly, the ERP extraction method reported by Lee, et al. [43] is based on one-unit independent component analysis (ICA) with reference. In general, the ICA is a powerful method of separating a mixture of signals into their underlying independent sources. In the context of ERP extraction, the ICA can identify and separate the independent components that are related to specific ERP components, such as P300 or N170. The usefulness of ICA for ERP extraction is that it separates the ERP components from the artifacts, such as eye blinks, eye movement, and muscle activity, which is relatively easy to extract ERP from background EEG signals. However, ICA is more computationally expensive and time-consuming than the PCA method, especially for large data or high-density multi-channel EEG. Both PCA and ICA methods could be used to extract ERP signals from the background EEG. However, compared to the proposed method in the current study, which jointly uses wavelet transformation and the Huffman coding method. The wavelet transform with a Bi-orthogonal B-Spline mother wavelet (bior3.5) can represent the whole ERP signal into a few wavelet coefficients, and unnecessary coefficients can be discarded without losing the critical stimulus-relevant information in the signal and maintaining the signal quality. The robustness of the proposed method is confirmed by comparing it with the previous studies utilizing the same dataset and classifiers used in this study.

## Limitations of the study

This study aims to propose an efficient and robust feature extraction method for single-trial ERPs analysis and classification of visual events. The study has a few limitations necessary to consider for future research on the subject matter. The first limitation could be using a two stimuli visual object detection task in the experiment. Thus, the results in this study address a binary classification problem only. A standard oddball task with three visual stimuli, including standard, target, and distracter stimuli, would be suitable for a multi-class problem. Second, the present study considered the whole duration of the ERPs trials starting from the stimuli onset to the stimuli offset. Suppose a specific component of ERP, for instance, P300 detection is desirable. Then the data should be analyzed in specific time windows, such as after 300ms of stimuli onset, in which case the proposed may not be useful because the length of the signal would be too short. Finally, the study considered all the electrodes in the classifiers, which might be a little expensive concerning computation costs. Thus, selecting specific electrodes, for instance, picking electrodes based on a 10–20 system or using only the midline electrodes (*Fz*, *Cz*, *Pz*, and *Oz*) where ERP peaks are more prominent in specific experiments would be appropriate. In addition, an automatic feature selection option could also be helpful, for instance, adopting principal component analysis (PCA) or Fisher’s Discriminant Ratio to reduce the number of features.

## Conclusion

This paper demonstrated an efficient and automatic method for single-trial ERP analysis and classification of visual events in the object detection task. The method is based on the optimum coefficients of the discrete wavelet transform and the Huffman coding technique. The performance of the proposed method is evaluated with real EEG experimental data for visual object detection tasks and showed robust feature extractions for single-trial ERPs from background noisy EEG and the classification of targets vs. standard trials with the machine learning classifiers. The proposed method allows studying single-trial visual evoked responses and discrimination of visual events. ERPs are an established way of conducting cognitive neuroscience research and related disciplines. Many research studies have reported using ERPs to study the brain’s functions, different states, and pathologies. The proposed method for single-trial ERPs analysis with a machine learning approach, such as ensemble use of discrete wavelet transform with the Huffman coding, would allow robust feature extraction for single trials classification and further would assist in the data analysis that may give new insights into studying the sensory and cognitive processes in the healthy and affected brain. This method may be implemented in the future for real-time systems, such as BCI applications for detecting and discriminating different motor and cognitive events.

## Data Availability

The experimental data can be accessed by requesting the corresponding author through email.
